# Family history of mood disorder weakens the association between personality traits and suicidality in depressed patients

**DOI:** 10.1192/j.eurpsy.2022.2172

**Published:** 2022-09-01

**Authors:** A. Kibitov, A. Nikolishin, N. Neznanov, G. Mazo, A. Kibitov

**Affiliations:** 1 Bekhterev National Medical Research Center for Psychiatry and Neurology, Translational Psychiatry Department, Saint-Petersburg , Russian Federation; 2 Federal State Budgetary Istitution Serbsky National Medical Research Centre for Psychiatry and Narcology of Ministry of Heath of the Russian Federation, Laboratory Of Molecular Genetics, Moscow, Russian Federation; 3 Bekhterev National Medical Center for Psychiatry and Neurology, Geriatric Psychiatry, Saint-Petersburg , Russian Federation; 4 Bekhterev National Medical Research Center for Psychiatry and Neurology, Translational Psychiatry Deartment, Saint-Petersburg , Russian Federation; 5 Serbsky National Medical Research Center on Psychiatry and Addictions, Molecular Genetics Laboratory, Moscow, Russian Federation

**Keywords:** Suicide, Depression, Family history

## Abstract

**Introduction:**

Depression is associated with a high risk of suicidal thoughts (ST) and behaviour (SB). Suicidality and depression have partially shared genetic underpinnings and family history of mood disorders (FH) can reflect genetic impact on specific features of depression. Thus, in depressed patients, FH may affect suicidality and its associations with other risk factors, such as personality traits.

**Objectives:**

We conducted a cross-sectional study to test the impact of FH on the association between suicidality and personality traits in depressed patients.

**Methods:**

200 depression in- and outpatients (64% (n=128) women, mean age (M(SD):36,21(15,09)) were enrolled. 28% (n=56) reported FH (“FH+” cohort), other patients comprised the “FH-” cohort. Columbia-Suicide Severity Rating Scale (C-SSRS) was used to assess ST and SB during the most suicidal period of life. Personality traits were assessed by Cloninger Temperament and Character Inventory (TCI-125). Information about FH and history of suicide attempts (SA) was obtained during the clinical interview.

**Results:**

Personality traits and suicidality characteristics (ST, SB, SA) did not differ between FH+ and FH- patients. In FH+, no differences in TCI-125 scores between suicide attempters and non-attempters were found, while in FH-, attempters had higher scores of TCI-125 “Novelty seeking” (p=0.002) and “Self-transcendence” (p=0.031) subscales. Multiple correlations between ST, SB and TCI-125 subscales were found only in FH-, In FH+, only one correlation (between ST and TCI-125 “Persistence” subscale (r=-0.288, p=0.038) was found.

**Conclusions:**

Our results showed a weakened association between personality traits and suicidality in depressed patients with FH of mood disorders, although more data on larger samples are needed.

**Disclosure:**

The study was supported by RSF grant # 20-15-00132

